# Towards an optimal diagnostic and prognostic model based on semi-quantitative assessment of ^18^F−FDG PET in children with autoimmune encephalitis

**DOI:** 10.3389/fimmu.2025.1457758

**Published:** 2025-04-02

**Authors:** Ziyuan Li, Jing Wu, Shuqi Wu, Shenrui Guo, Mingming Cao, Weiwei Cheng, Hui Wang, Ling Li, Yafu Yin

**Affiliations:** ^1^ Department of Nuclear Medicine, Xinhua Hospital Affiliated To Shanghai Jiao Tong University School of Medicine, Shanghai, China; ^2^ Department of Pediatric Neurology, Xinhua Hospital Affiliated To Shanghai Jiao Tong University School of Medicine, Shanghai, China

**Keywords:** autoimmune encephalitis, children, positron emission tomography, semi-quantitative, prognosis

## Abstract

**Purpose:**

The metabolic pattern in autoimmune encephalitis (AE) has been frequently reported. Through this semi-quantitative analysis, we aim to explore a practical diagnostic model based on positron emission tomography (PET) for timely diagnosis of pediatric AE with high accuracy. Moreover, we aim to identify factors that affect the prognosis of pediatric AE and explore the utility of PET as a prognostic biomarker.

**Method:**

Data were collected from 93 AE patients and 67 non-AE patients (age range: 1-18 years old). Semi-quantitative parameters of ^18^F-FDG PET imaging were evaluated, including the score of cortical lesion extent and the ratios of lesion-to-basal ganglia and thalamus. The Clinical Assessment Scale in Autoimmune Encephalitis (CASE) was used to rate the disease severity and long-term outcome. Multivariate statistical analysis was used to establish a diagnostic model and analyze predictors.

**Results:**

The diagnostic model includes three PET parameters. The sensitivity, specificity, and accuracy of the model are 91.4%, 85.1%, and 88.8%, respectively. Participants were followed up for a median of 34 months. Logistic regression analysis indicated that male, initial CASE score >4.5,memory dysfunction, and the ratio of the maximum SUV of the lesion to thalamus (SUVRmax_L/T_) < 0.577 are independent factors associated with poor prognosis in AE. We established a prognostic model through these predictors.

**Conclusion:**

^18^F-FDG PET plays a vital role in the diagnosis and prognosis of AE. The PET-based diagnostic model has higher specificity and accuracy than visual analysis. The prognostic model is a useful predictive tool for the long-term prognosis of children with AE.

## Highlights

- Question: How to diagnose and predict the prognosis of autoimmune encephalitis in children through PET?
- Pertinent findings: Through the semi-quantitative analysis of PET, a diagnostic and prognostic model including several semi-quantitative indexes was established.
- Implications for patents care: The two models can help the early diagnosis and prognosis of childhood autoimmune encephalitis, respectively

## 1 Introduction

Autoimmune encephalitis (AE) is a group of central nervous system (CNS) inflammatory disorders mediated by autoimmune mechanisms. It primarily affects children and young adults (1), with a rate of 11.6 per 100,000 children (2). Timely and accurate diagnosis and early initiation of immunotherapy are critical for children with AE, for which delayed treatment results in poor outcomes (3). However, misdiagnosis of AE is not uncommon in many institutions, including specialized centers (4). It has been highlighted that a broad differential diagnosis should be considered in AE diagnosis (4, 5). The exclusion of alternative disorders is crucial for the AE diagnostic criteria (6, 7).

PET has a unique value in the diagnosis of AE (8). Clinical approaches to the diagnosis of AE in pediatric patients recommend considering PET if available and/or if required based on initial investigation (2). We have reported typical findings of ^18^F-FDG PET/CT in children with AE in a prospective study (9). This time, we attempted to conduct a semi-quantitative analysis of FDG PET in the differential diagnosis of pediatric AE.

Studies to identify prognostic biomarkers of AE are needed. PET can be used as a prognostic test (10). There have been several studies of PET in adult AE outcomes, but most of them have been limited to specific antibodies (11, 12). Pediatric AE often differs from adult-onset AE in symptoms and ultimate prognosis (13). The prognosis of pediatric AE remains to be further investigated. Here, we performed a retrospective study to seek prognostic biomarkers for pediatric AE and explore the validity of PET in AE prognosis.

## 2 Methods

### 2.1 Patients selection

This retrospective analysis was conducted from an observational prospective study (Clinical Trials.gov. NCT02969213). The patients who met the following criteria were included (1): who were suspected of AE and younger than 18 years old, older than 1 year old; (2) FDG PET imaging was performed from May 14, 2019, to August 30, 2022, in Xinhua Hospital affiliated to Shanghai Jiao Tong University School of Medicine. Exclusion criteria were listed: (1) who were with other neuropsychiatric disorders; (2) others that could affect the semi-quantitative analysis of FDG PET imaging. According to the criteria mentioned in our previous report (9), the children diagnosed with AE were included in the AE group. Children who were ultimately not diagnosed with AE were included in the non-AE group.

### 2.2 Clinical and paraclinical data

Clinical and paraclinical information was recorded by searching the electronic medical records. The scale, named the Clinical Assessment Scale for Autoimmune Encephalitis (CASE), was used to rate the severity of AE. CASE scores were assessed at admission for all patients. The CASE includes nine symptoms: seizures, memory dysfunction, psychiatric symptoms, consciousness, language problems, dyskinesia/dystonia, gait instability and ataxia, brainstem dysfunction, and weakness. Each item should be scored from 0 to 3 according to the severity of symptoms, with a total maximum score of 27 (33). The nine variables included in the CASE were obtained through medical records at admission.

### 2.3 Clinical follow-up

The follow-up period from discharge until follow-up comprised at least 12 months for all AE patients. CASE scores to assess long-term outcomes were obtained by telephone follow-up. AE patients with follow-up data were divided into two groups according to their CASE scores at follow-up. Referring to the grouping criteria in a previous article (14), we classified patients with CASE scores of 0-4 at follow-up into the good outcome group and those with scores greater than 4 into the poor outcome group.

### 2.4 Brain ^18^F−FDG PET/CT imaging

^18^F-FDG PET scanning was acquired using a Siemens Biograph mCT-64 scanner (Erlangen, Germany). All patients fasted for at least 4 hours and rested in a quiet, dim room before injection. ^18^F-FDG was intravenously injected at a dose of 3.7MBq/kg. A 10-minute brain PET/CT scan was performed about 50 minutes after injection.

### 2.5 Imaging analysis

Two nuclear medicine doctors with extensive experience in brain FDG PET/CT imaging visually evaluated all PET/CT images. In case of disagreement, a third senior physician made the judgment. The criteria for FDG PET/CT diagnosis of AE: large lobar hypometabolism with or without focal hypermetabolism found on PET/CT was defined as AE. Large lobar hypometabolism means extensive decreased glucose metabolism in more than one lobe or one lobe with bilateral involvement. We recorded the metabolic alterations of cortical lesions, the basal ganglia, and the thalamus.

### 2.6 Semi-quantitative analysis

Firstly, a score of cortical lesion extent was used to reflect the extent of cortical lesion: one point was recorded for a lesion that involved either lobe unilaterally. The total score was eight. Secondly, we measured the standard uptake value (SUV) of the most significant hypometabolic lesion, the basal ganglia and thalamus. If there was no significant hypometabolic lesion, the SUV of the parietal cortex was measured instead. Using Siemens TrueD software from Germany, the ROI was delineated with a threshold of 40% SUVmax, and the cortical lesion was delineated within a range of approximately 200 mm^2^. For the basal ganglia and thalamus, manually draw circular 3D ROIs of appropriate diameter in the corresponding areas of the PET image cross-section. Software automatically measures SUVs of ROI. We took the average of SUVs of the basal ganglia and thalamus on both sides. Lastly, metabolic ratios, including lesion to basal ganglia and lesion to thalamus, were constructed separately by SUVmax, SUVmean, and SUVmin. SUVRmax_L/B_ and SUVRmax_L/T_ stand for the ratio of the maximum SUV of the lesion to the basal ganglia and the lesion to the thalamus. SUVRmean_L/B_ and SUVRmean_L/T_ stand for the ratio of the mean SUV of the lesion to the basal ganglia and the lesion to the thalamus. SUVRmin_L/B_ and SUVRmin_L/T_ mean the ratio of the minimum SUV of the lesion to the basal ganglia and the lesion to the thalamus.

### 2.7 Statistical analysis

SPSS 26.0 software (IBM Corp.) and Prism 9 (GraphPad software) were used for statistical analyses. The Shapiro-Wilk test was used to determine whether the data distribution for continuous variables was normal. Continuous variables were compared using the *t*-test or the non-parametric Mann-Whitney *U*-test. Categorical variables were analyzed by Chi-squared tests and Fisher’s exact tests. Multivariate binary logistic regression analysis was employed to construct the diagnostic models and derive factors associated with long-term outcomes. Receiver-operating characteristic curves (ROC) were used to calculate cut-off values and assess the discrimination ability of semi-quantitative parameters and diagnostic models. A two-tailed *p*-value less than 0.05 (*p* < 0.05) was considered statistically significant.

## 3 Results

### 3.1 Clinical and paraclinical characteristics

In total, 160 patients met the inclusion criteria, of which 93 patients were diagnosed with AE and 67 with non-AE. The baseline demographic, clinical and paraclinical data were shown in **Table 1**. Of the AE patients, 30 were antibody-positive. 10 patients had NMDAR antibodies(1 in serum,8 in CSF,1 in serum and CSF). 5 patients had GQ1b antibodies in serum. Other antibodies positive in serum included Ri(2),MOG(2), Amphiphysin(2),CASPR2(1),Ma2(1),Recoverin(1),AMPAR1(1), Hu combined with Amphiphysin(1), NMDAR combined with mGluR5(2) and SOX1 combined with GQ1b(1). 1 patient had DPPX antibody positive in CSF. Final diagnoses in the non-AE group included viral encephalitis, epilepsy, developmental delay, narcolepsy, etc. None of the children showed specific features of AE on MRI.

**Table 1:** Demographic, clinical and paraclinical characteristics.

	AE (n=93)	Non-AE (n=67)	*p*-Value
**Age (yrs.), mean [range]**	7.3 [1-17]	7.6 [1-15]	0.813
**M/F**	47/46	41/26	0.589
**Duration of symptom onset (month), mean [range]**	18.5 [0.25-104.3]	28.4 [0.29-120]	0.069
**Initial CASE score, mean [range]**	4 [0-9]	3 [0-6]	0.0003**
Symptoms, n (%)			
Seizures	53 (57.0%)	42 (62.7%)	0.469
Memory dysfunction	11 (11.8%)	2 (3.0%)	0.043*
Movement disorders	33 (35.5%)	11 (16.4%)	0.008**
Neuropsychiatric symptoms	37 (39.8%)	12 (17.9%)	0.003**
Sleep alteration	21 (22.6%)	10 (14.9%)	0.227
Language barriers	33 (35.5%)	12 (17.9%)	0.015*
**CSF abnormalities, n (%)**	46 (49.5%)	4 (6.0%)	<0.0001***
**EEG abnormalities, n (%)**	58 (62.4%)	42 (62.7%)	0.967

* means P<0.05, ** means P<0.01, ***means P<0.0001.

### 3.2 Diagnostic performance by visual analysis

According to the criteria for FDG PET/CT diagnosis of AE, 129 patients were diagnosed as AE, of which 89 were truly AE. Four AE patients were misdiagnosed as non-AE. 40 non-AE patients were misdiagnosed as AE. The sensitivity, specificity, and accuracy of visual diagnosis were 95.7% (89/93), 40.3% (27/67), and 72.5% (116/160), respectively. The sensitivity of visual diagnosis is good, but the specificity is low.

### 3.3 AE diagnostic model based on FDG-PET

**Figure 1:**
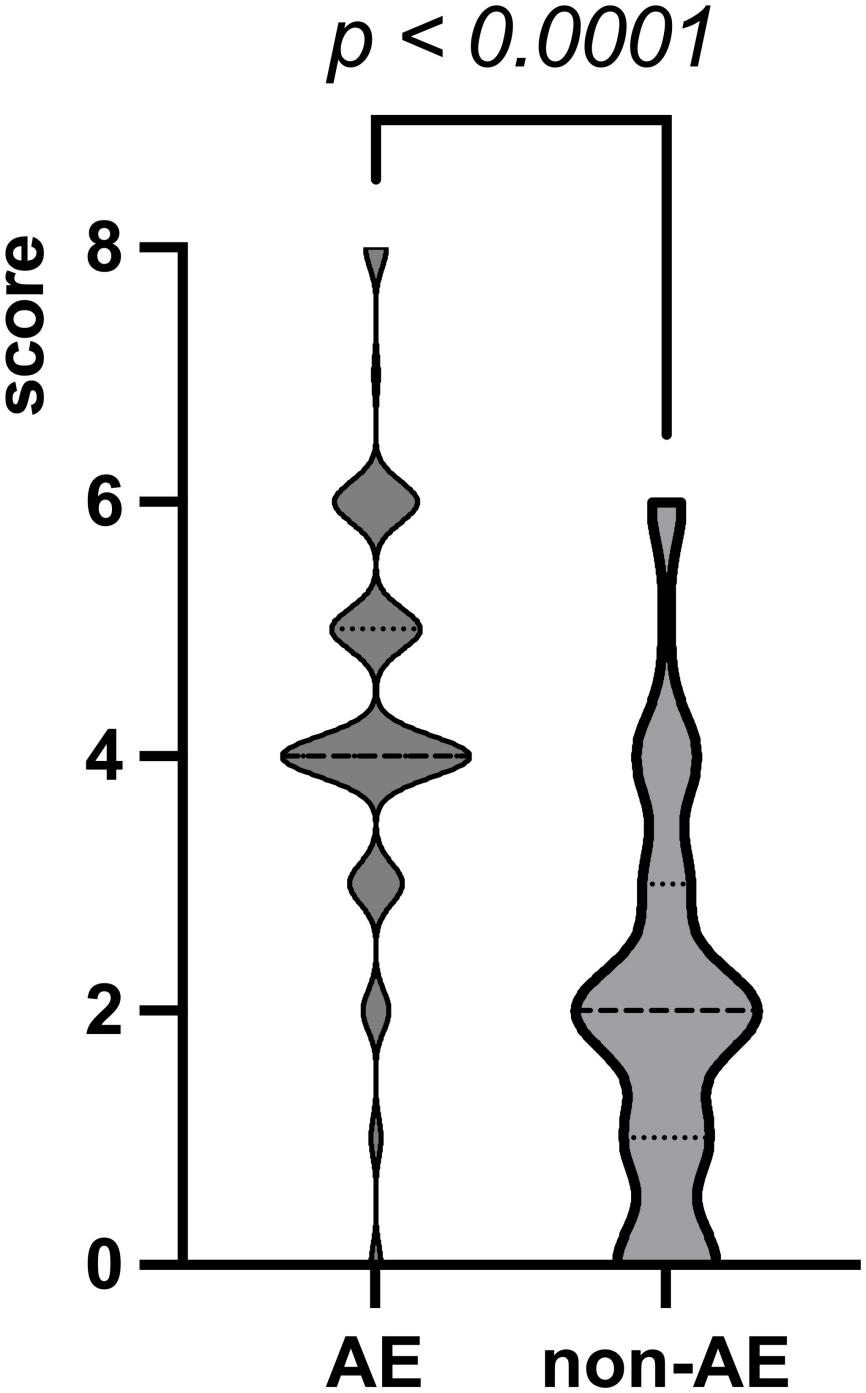
The score of cortical lesion extent. The max, mean, and min values in the AE group are 8, 4, and 0, respectively. The max, mean, and min values in the non-AE group are 6, 2, and 0, respectively.

The cortical lesions in the AE group were more extensive than those in the non-AE group. The score of cortical lesion extent in the AE group was significantly higher (**Figure 1**). At the optimal cut-off of 2.5, sensitivity was 90.3%, specificity was 71.6% and AUC was 0.810.

**Figure 2:**
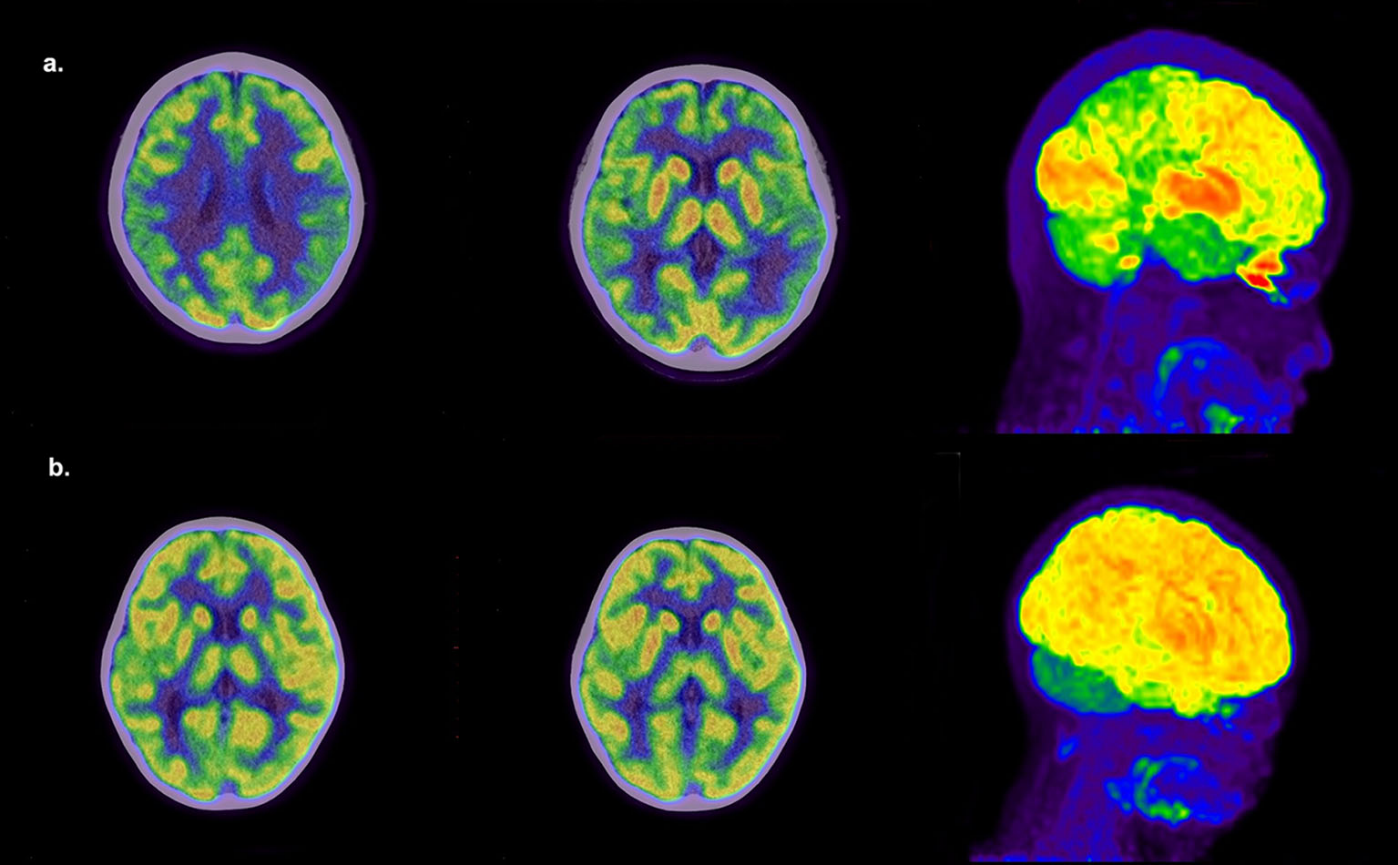
Representative PET images. **(a)** An 11-year-old girl with AE had a 1-week history of seizures. ^18^F-FDG PET/CT shows extensive hypometabolism in bilateral frontal, parietal, and temporal lobes and hypermetabolism in the basal ganglia and thalamus. **(b)** A 3-year-old girl with a 2-year history of seizures and developmental delay was finally diagnosed with intractable epilepsy. ^18^F-FDG PET/CT shows hypometabolism in the right temporal lobe and right thalamus.

There were also significant differences in metabolic changes between the two groups. **Figure 2** shows typical PET images of AE and non-AE. The AE group showed a higher proportion of hypometabolism in the cortex and hypermetabolism in the basal ganglia and thalamus (**Table 2**). We constructed metabolic ratios using SUVs of cortical lesions, basal ganglia, and thalamus and figured out whether these ratios could be used to distinguish AE from non-AE. The diagnostic performance of these ratios is presented in **Table 3**.

**Table 2:** Metabolic alteration in two groups.

	AE (n=93)	Non-AE (n=67)	*p*-Value,Χ^2^
Cortex, n (%)			<0.0001***,19.190
Only hypometabolism	80 (86.0%)	47 (70.1%)	
Only hypermetabolism	0 (0.0%)	3 (4.5%)	
Hypermetabolism and hypometabolism	11 (11.8%)	4 (6.0%)	
Normal metabolism	2 (2.2%)	13 (19.4%)	
Basal ganglia, n (%)			<0.0001***,25.552
Hypermetabolism	45 (48.4%)	7 (10.4%)	
Normal metabolism	48 (51.6%)	60 (89.6%)	
Thalamus, n (%)			<0.0001***,19.861
Hypometabolism	3 (3.2%)	11 (16.4%)	
Hypermetabolism	20 (21.5%)	1 (1.5%)	
Normal metabolism	70 (75.3%)	55 (82.1%)	

***means P<0.0001.

**Table 3:** Diagnostic performances of ratios.

Ratios	AE (n=93)	Non-AE (n=67)	AUC	cut-off value	*p*-value
SUVRmax_L/B_	0.54 ± 0.10	0.70 ± 0.13	0.827	0.615	<0.0001***
SUVRmax_L/T_	0.66 ± 0.11	0.86 ± 0.16	0.828	0.757	<0.0001***
SUVRmean_L/B_	0.75 ± 0.16	0.96 ± 0.17	0.768	0.844	<0.0001***
SUVRmean_L/T_	0.88 ± 0.17	1.11 ± 0.19	0.796	0.970	<0.0001***
SUVRmin_L/B_	2.34 ± 0.66	3.05 ± 0.85	0.718	2.759	<0.0001***
SUVRmin_L/T_	2.35 ± 0.79	3.08 ± 0.84	0.725	2.659	<0.0001***

***means P<0.0001.

Five parameters, score of cortical lesion extent, SUVRmax_L/B_, SUVRmax_L/T_, SUVRmean_L/B_, and SUVRmean_L/T_, showed a higher AUC between the two groups. Therefore, we finally select these 5 parameters for further analysis. Before multivariate logistic regression analysis, all continuous variables were converted to binary variables according to their respective cut-off values (**Supplementary Table 1**). Three parameters were selected by logistic regression to establish a diagnostic model to distinguish between AE and non-AE.

**Figure 3:**
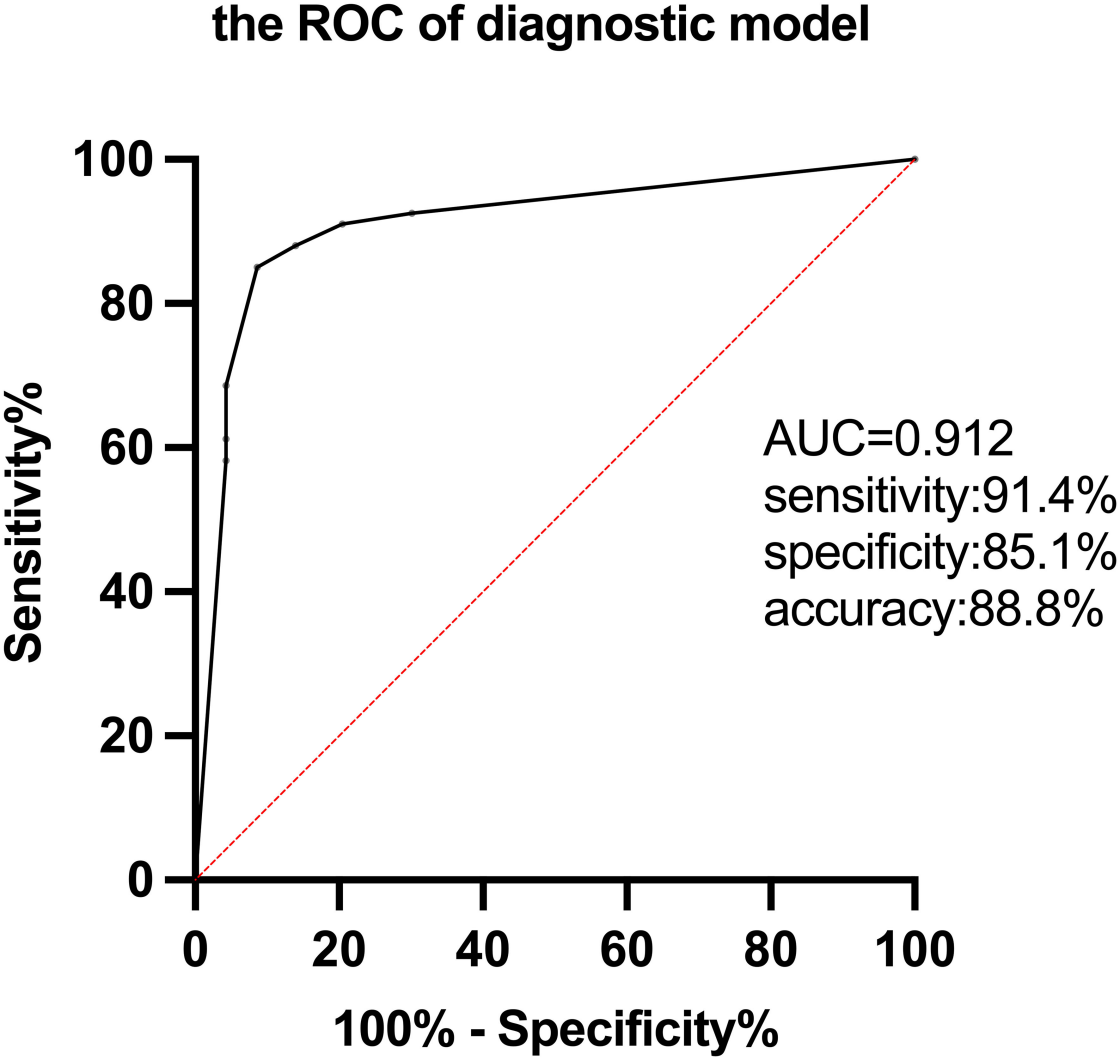
The ROC of the diagnostic model for AE based on FDG-PET semi-quantitative parameters.

Multivariate regression analysis demonstrated that the combination of the score of cortical lesion extent (*p* < 0.0001), SUVRmax_L/B_ (*p* = 0.007), and SUVRmax_L/T_ (*p* = 0.001) were independently correlated with AE. An associated diagnostic model for AE was constructed as follows $Logit\left[P\;=\;AE\right]=\;2.102\left[the\;score\;of\;cortical\;lesion\;extent\right]+1.483\;\left[SUVRmaxL/B\right]\;+\;1.801SUVRmaxL/T]-\;2.719$ . The ROC curve confirmed that the diagnostic model could markedly distinguish AE from non-AE. The sensitivity, specificity, accuracy, and AUC value were 91.4%, 85.1%, 88.8%, and 0.912, respectively(**Figure 3**).

### 3.4 Long-term outcomes of AE patients

**Figure 4:**
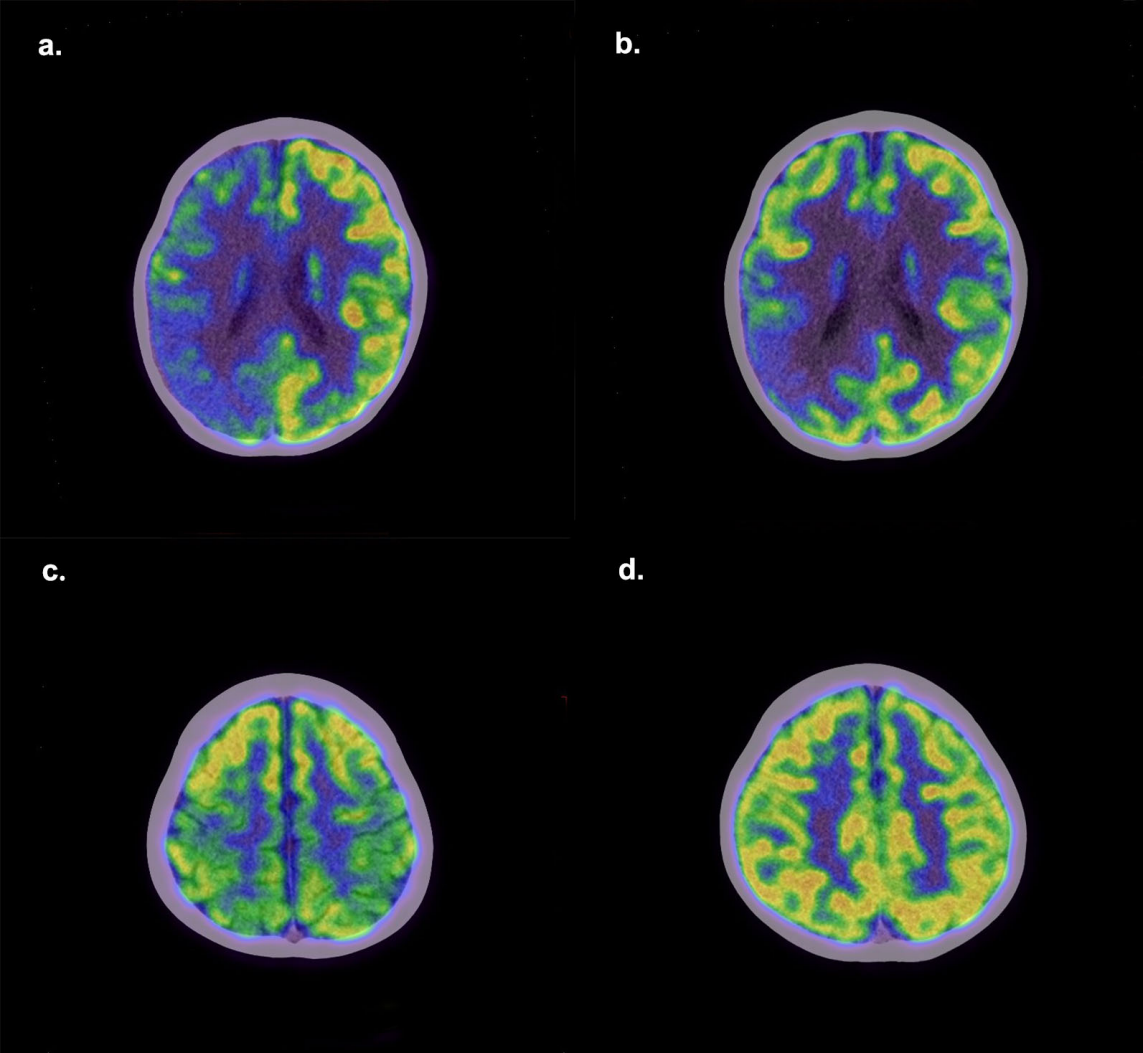
Representative PET images of patients with poor and good outcomes. Upper: A boy with AE had poor epilepsy control. His initial CASE score was 5, and three years after discharge, his CASE score remained 5. **(a)** The first 18F-FDG PET shows hypometabolism in the right cerebral cortex. **(b)** One year later, the second ^18^F-FDG PET shows there is still a seriously hypometabolic region in the right parietal lobe. Bottom: A boy had a 1-year history of gait instability and a 1-month history of severe language problems. Two years later, his language function has returned to normal, but gait in stability still exists. His CASE score has changed from 5 to 1. **(c)** The first ^18^F-FDG PET shows hypometabolism in bilateral parietal lobes. **(d)** Three months later, the second ^18^F-FDG PET shows the metabolism in the parietal lobe has recovered to normal.

**Figure 5:**
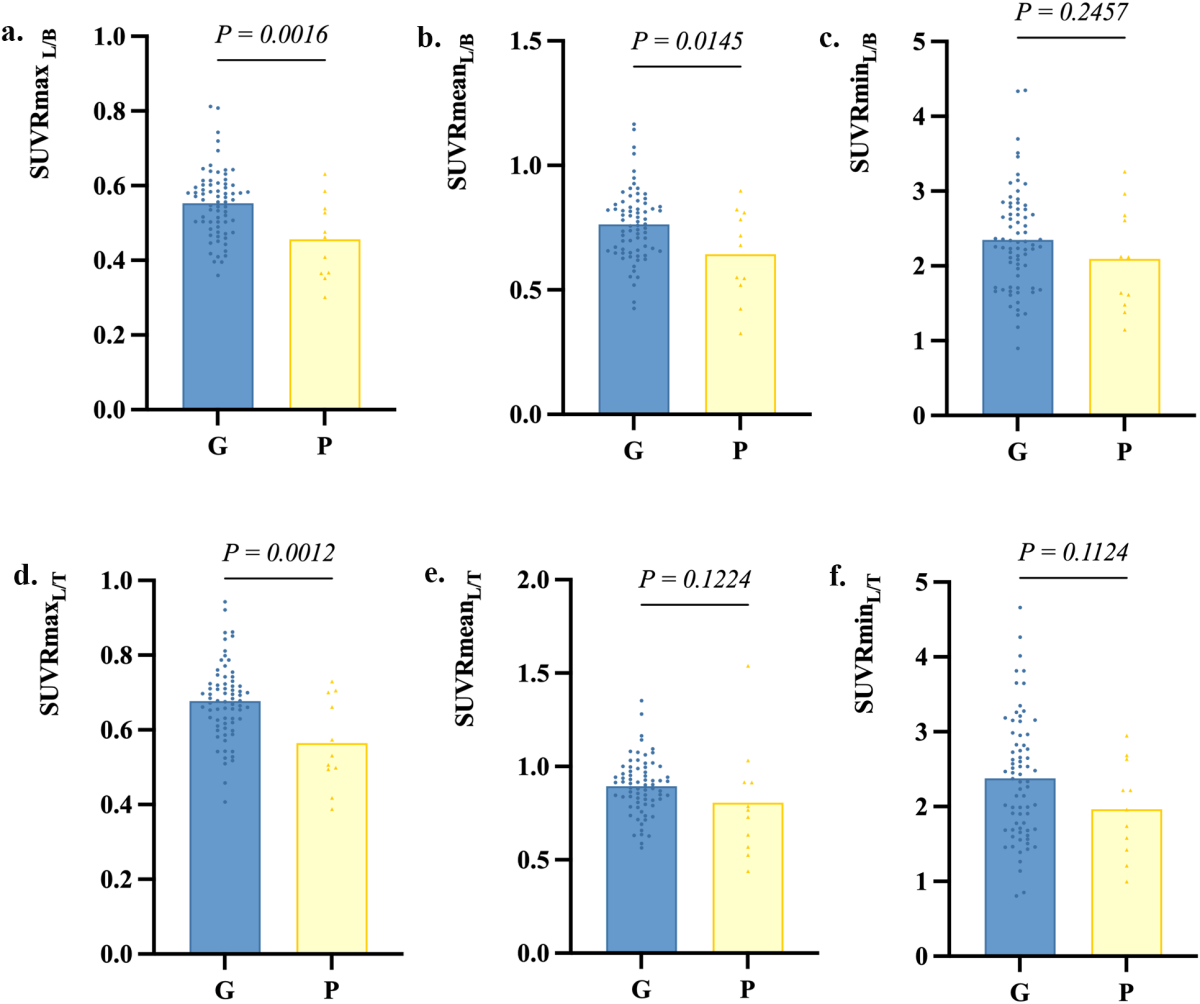
Metabolic ratios in the good outcome group and poor outcome group. G, the good outcome group; P, the poor outcome group.

Follow-up data were available for 83 AE patients with a median follow-up duration of 34 (12-51) months, with no significant differences between the poor outcome group and the good outcome group regarding follow-up duration. Patients with follow-up CASE score >4 (n=11) were divided into the poor outcome group, and the rest (n=72) were divided into the good outcome group. **Figure 4** shows PET images of two patients from the good and poor outcome groups. Three metabolic ratios tended to be significantly lower in the poor outcome group (*p*<0.05) (**Figure 5**), and their cut-off values were calculated by ROC analysis. The clinical and paraclinical data between the two groups were compared by univariate analysis (**Table 4**). Univariate analysis indicated that there were significant differences between the good and poor outcome groups in sex, initial CASE>4.5, memory dysfunction and three metabolic ratios (SUVRmax_L/B_, SUVRmax_L/T_, SUVRmean_L/B_).

**Table 4:** Univariate analysis to compare clinical and paraclinical data between the good and poor outcome groups.

Factors	Good outcome (n=72)	Poor outcome (n=11)	*p*-Value	OR
Age (yrs.), mean [range]	7.4 [1-17]	7.2 [1-10]	0.870	0.984
M/F,	30/39	10/1	0.022*	11.818
Duration of symptom onset (month), mean [range]	18.8 [0.25-72]	17.7 [0.6-78.2]	0.865	0.997
Initial CASE > 4.5, n (%)	23 (31.9%)	10 (90.9%)	0.007**	1.641
Seizure, n (%)	39 (54.2%)	7 (63.6%)	0.558	1.481
Memory dysfunction, n (%)	5 (6.9%)	3 (27.3%)	0.049*	5.025
Movement disorders, n (%)	26 (36.1%)	5 (45.5%)	0.552	1.474
Neuropsychiatric symptoms, n (%)	30 (41.7%)	3 (27.3%)	0.369	0.525
Sleep alteration, n (%)	17 (23.6%)	1 (9.1%)	0.298	0.324
Language barriers, n (%)	24 (33.3%)	5 (45.5%)	0.436	1.667
CSF abnormalities, n (%)	37 (51.4%)	5 (45.5%)	0.714	0.788
EEG abnormalities, n (%)	45 (62.5%)	7 (63.6%)	0.942	1.050
Second-line treatment, n (%)	10 (13.9%)	2 (18.2%)	0.707	0.726
Score of the cortical lesion, mean [range]	4 [0-8]	4 [1-6]	0.533	2.000
SUVRmax_L/B_, mean ± SD	0.55 ± 0.09	0.46 ± 0.11	0.008**	6.125
SUVRmax_L/T_, mean ± SD	0.68 ± 0.10	0.56 ± 0.12	0.001**	10.850
SUVRmean_L/B_, mean ± SD	0.76 ± 0.14	0.64 ± 0.18	0.001**	14.167

* means P<0.05, ** means P<0.01.

### 3.5 Predictors of prognosis and a prognostic model

All factors with a *P*-value < 0.05 in **Table 4** were included in a multivariate logistic regression model (**Supplementary Table 2**). Multivariate logistic regression analysis showed that male ([OR] =44.74, *p* = 0.012), initial CASE>4.5 ([OR] =37.08, *p* = 0.012), memory dysfunction ([OR] =22.90, *p* = 0.047), and SUVRmax_L/T_ < 0.577 ([OR] =45.00, *p* = 0.005) were independent risk factors associated with poor prognosis of AE. The β value for each factor was obtained by logistic regression. We took the smallest β value (3.131) as a basic unit and scored it as 1. According to the respective β values of each factor, we got optimal new points. A practical predictive model was built based on the optimal new points (**Table 5**). For the optimized prognostic model, the AUC was 0.948 (95% CI, 0.896 to 1.000). With a cut-off of 1.5, the predicted sensitivity was 100%, and the specificity was 75%. With a cut-off of 2.5, the predicted sensitivity was 63.6%, and the specificity was 98.6%.

**Table 5:** The factors and values of the optimized prognostic model.

Prognostic factors	β	Optimal point
Sex	3.801	
female		0
male		1
Initial CASE	3.613	
<4.5		0
>4.5		1
Memory dysfunction	3.131	
without		0
with		1
SUVRmax_L/T_	3.807	
≥0.577		0
<0.577		1
Total score		0-4

## 4 Discussion

The diverse clinical features of AE can mimic a variety of other neurological disorders, making a timely and proper clinical diagnosis difficult (15). Although brain MR imaging is regarded as the first choice of imaging test, ^18^F-FDG PET/CT has been proven to have a higher sensitivity than MR in AE diagnosis (8, 16). More than half of patients with AE showed non-specific features in MR (16). In our study, all the AE children presented normal or non-specific features in MR, as mentioned in our previous study (9), one reason is that the children with specific features of encephalitis have been diagnosed and treated without the need for FDG PET. PET is valuable in MR-negative patients and can provide vital information to aid in the clinical diagnosis. Moreover, PET findings are more associated with clinical symptoms, disease severity, and recovery after therapy than the MR findings (17). A study concluded that there are two different PET scan patterns in AE. One is a mixture of hyper- and hypometabolism, the other is diffusely reduced cortical uptake of ^18^F-FDG ^(^ 18). A diffuse cortical hypometabolism has been found in encephalitis with different antibody types (19, 20). Our previous research concluded that large lobar hypometabolism with or without focal hypermetabolism is the FDG PET/CT diagnostic criteria for AE (9). But visual diagnosis caused low specificity. The reason for this is that some non-AE patients exhibit large lobar hypometabolism, which is the standard for AE visual diagnosis. Part of non-AE patients are ultimately diagnosed with epilepsy. The interphase of epilepsy is characterized by low metabolism of the lesion. A study has shown that the proportion of children with epilepsy with multi lobe hypometabolism is about 47% (21). Therefore, the specificity of simple visual analysis is low. The hypermetabolism in basal ganglia was frequently reported in AE (22, 23). Metabolic alterations in the thalamus have also been reported (24, 25), although not in large numbers. In our study, hypermetabolism in basal ganglia was present in 48.4% of AE patients, and diffuse cortical hypometabolism was in 86%. We quantified these PET features into reproducible diagnostic parameters. The diagnostic model containing semi-quantitative indicators improves diagnostic specificity. Many semi-quantitative analyses rely on software that is not convenient for clinical use (11, 26). We referred to this research (27) and used a simple measurement method to establish the diagnostic model with high sensitivity and accuracy, and the three parameters in the model are easily obtained.

Future studies have been suggested to focus on the evaluation of biomarkers as potential predictors of AE outcomes (28). Most studies in AE used the modified Rankin scale (mRS) as an outcome measure. However, mRS is insufficient to evaluate the neurological improvements of AE as a scale of global disability for acute stroke patients (29). The CASE has good reliability and validity in AE (30–32).It can reflect neuropsychological status, perform satisfactorily even in AE patients with mild symptoms, and changes in CASE score are more sensitive to changes in severity than mRS. The CASE score greater than 9 in pediatric AE patients suggests the need for second-line therapy, and ICU admission may be necessary when the CASE score is greater than 14 (30). We found that an initial CASE score above 4.5 was a risk factor for a poor prognosis. This means that the severity of symptoms is associated with prognostic outcomes. The lower severity of symptoms assessed as no need for ICU support predicts a good outcome (33), which is similar to our results.

PET is one of the possible prognostic biomarkers (10). Cortical hypometabolism may be a predictor of poor outcome or relapse in anti-NMDAR-encephalitis in children (20). Compared with the patients with a good outcome, the patients with a worse long-term outcome showed lower metabolic activity in particular cortical regions at baseline, including the orbital frontal and cingulate gyrus (11). Our finding indicated that the ratios of the most significant hypometabolic lesion to basal ganglia and thalamus are lower in the poor outcome group. Baseline SUVRmax_L/T_ less than 0.577 may be a marker for a poor prognosis. ^18^F-FDG-PET imaging has the potential to improve the estimation of follow-up evaluation and therapy monitoring in patients with AE (17, 34). Among the patients included in this study, 13 AE patients underwent PET reexamination, and 10 of them had follow-up information. In the good outcome group, some patients showed a reduction in the range of cortical lesions on PET follow-up after treatment, while some patients had elevated basal ganglia metabolism compared to before treatment. In the poor outcome group, the patient’s second PET showed a similar or slightly reduced range of cortical lesion as before, but there were still lesions with very low metabolism present. Due to the small number of cases and varying intervals between two PET examinations, it is not suitable for statistical analysis. Further research is needed to determine whether changes in PET after treatment can indicate prognosis.

Prognostic studies in adults with AE have found that memory dysfunction is associated with poor prognosis (35). This may be due to reduced connectivity of the anterior hippocampus and the anterior default mode network. These connections seem most vulnerable, which may cause poor memory recovery (36). Other symptoms mentioned in existing studies that may be associated with a poor prognosis for AE in children include disturbance of consciousness, limb dyskinesia (37), altered consciousness, and central hypoventilation (38, 39). As for sex, a study found boys were significantly more likely to need a longer hospital day and a longer course of steroid. Boys might have a worse outcome than girls (40). We found that male is a predictor of poor outcomes, too. While another study indicates outcomes were similar for females and males (41). For patients with anti-NMDA receptor encephalitis, second-line immunotherapy was significantly associated with better outcomes (33). We found that patients treated with second-line therapy had more severe symptoms on admission but recovered well and could return to full recovery. However, our study has not yet found a significant difference between the effects of first-line and second-line treatment on prognosis.

However, this study has some limitations. First, we construct a diagnostic model based on the semi-quantitative parameters of FDG PET from 160 patients, which is not a large data set and lacks validation data. And multi-center studies are necessary to validate the model. For prognostic model, small data, especially the poor outcome group with few patients is the most significant shortcoming, and clinical factors enrolled for analysis are not sufficient, such as the initial time and duration of therapy are not included. Further study enrolled more cases would be performed in future.

## 5 Conclusion

Compared with visual analysis, the diagnostic model based on metabolic ratios of FDG PET has better specificity and accuracy. Male, initial CASE>4.5, memory dysfunction, and SUVRmax_L/T_<0.577 may have predictive value for the poor outcomes of pediatric AE. We provide practical models for diagnosis and prognostic prediction of AE in children. Our results confirm the value of FDG PET in the diagnosis and prognosis of AE.

## Data Availability

The raw data supporting the conclusions of this article will be made available by the authors, without undue reservation.
