# Supplementary material for: Towards an optimal diagnostic and prognostic model based on semi-quantitative assessment of 18F−FDG PET in children with autoimmune encephalitis
Source: Front Immunol. 2025 Apr 2;16:1457758. doi: 10.3389/fimmu.2025.1457758 (PMC12000777; doi:10.3389/fimmu.2025.1457758)
Supplement: Supplementary file 1 [file Table1.docx]

**Supplementary table 1**. PET factors analyzed in multiple logistic regression analysis for diagnostic model

| Factors | Definition |
| --- | --- |
| The score of cortical lesion range |  |
| >2.5 | 1 |
| <2.5 | 0 |
| L/B_SUVRmax_ |  |
| <0.615 | 1 |
| ≥0.615 | 0 |
| L/T_SUVmax_ |  |
| <0.757 | 1 |
| ≥0.757 | 0 |
| L/B_SUVRmean_ |  |
| <0.844 | 1 |
| ≥0.844 | 0 |
| L/T_SUVRmean_ |  |
| <0.970 | 1 |
| ≥0.970 | 0 |

**Supplementary table 2**. Factors analyzed in multiple logistic regression analysis for prognostic model

| Factors | Definition |
| --- | --- |
| Sex |  |
| male | 1 |
| female | 0 |
| Initial CASE |  |
| >4.5 | 1 |
| <4.5 | 0 |
| Memory dysfunction |  |
| with | 1 |
| without | 0 |
| L/B_SUVRmax_ |  |
| <0.480 | 1 |
| ≥0.480 | 0 |
| L/T_SUVRmax_ |  |
| <0.577 | 1 |
| ≥0.577 | 0 |
| L/B_SUVRmean_ |  |
| <0.552 | 1 |
| ≥0.552 | 0 |
